# Encoding function into polypeptide-oligonucleotide precision biopolymers†
†Electronic supplementary information (ESI) available. See DOI: 10.1039/c8cc04725a


**DOI:** 10.1039/c8cc04725a

**Published:** 2018-09-26

**Authors:** Weina Liu, Felix Boldt, Yu Tokura, Tao Wang, Bikram Keshari Agrawalla, Yuzhou Wu, Tanja Weil

**Affiliations:** a Max-Planck-Institute for Polymer Research , Ackermannweg 10 , 55128 Mainz , Germany . Email: weil@mpip-mainz.mpg.de; b Department of Inorganic Chemistry I , Ulm University , Albert-Einstein-Allee 11 , 89081 Ulm , Germany; c School of Materials Science and Engineering , Southwest Jiaotong University , 610031 , Chengdu , China; d Hubei Key Laboratory of Bioinorganic Chemistry and Materia Medica , School of Chemistry and Chemical Engineering , Huazhong University of Science and Technology , Luoyu Road 1037 , 430074 Hongshan , Wuhan , P. R. China . Email: wuyuzhou@hust.edu.cn

## Abstract

We report a novel synthesis strategy to prepare precision polymers providing exact chain lengths, molecular weights and monomer sequences that allow post modifications by convenient DNA hybridization. Two grafted single strand DNA (ssDNA) side chains serve as a versatile platform for sequence-specific attachment of chromophores, proteins, cell-targeting peptide, and a Y-shape DNA linker. This approach resembles a LEGO®-type incorporation of functionalities to create functional biopolymers of high structure definition under mild conditions.

## Abstract 2

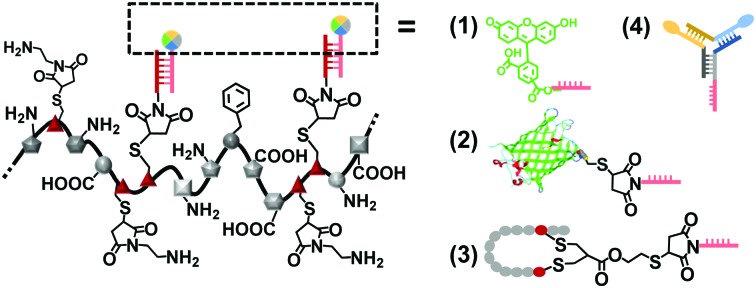
We report a novel synthesis strategy to prepare precision polymers providing exact chain lengths, molecular weights and monomer sequences that allow post modifications by convenient DNA hybridization.

## 

The synthesis of polymers consisting of precisely defined sequences and molecular weights has been one of the holy grails in polymer chemistry. Nature, as a role model, produces monodispersed macromolecules such as polypeptides, proteins, and DNA providing distinct monomer sequences that determine their structures, interactions and functions.^1^ In contrast, traditional polymerization reactions inevitably produce polymers with sequence heterogeneity and polydispersity. The synthesis of defined polymeric architectures has long been limited to highly branched dendrimers, where information was introduced into the dendritic arms of the different generations.^2^ However, in the past years, there has been remarkable progress in optimizing sequence-controlled chain-growth or step-growth polymerizations producing polymers with very narrow molecular weight distributions and controlled sequences.^3^ Templated polymer synthesis that made use of the controlled assembly of peptide nucleic acids or polyaniline clearly made a large step forward to realize sequence and molecular weight precision.^4^ However, there is still no synthetic strategy available that can compare to nature's unique capabilities in combining diverse functionalities and structural perfection.

On the other hand, advances in bioengineering yield recombinant proteins^5^ and protein–polymer conjugates^6^ with rationally designed sequences and structures, which have been denoted as “monodisperse biopolymers”. Following a different approach, we have reported the concept of converting the native protein human serum albumin (HSA) into narrowly dispersed polyamide copolymers by step-wise denaturation and grafting of polyethylene(oxide) (dHSA-PEO).^7^ Such protein-derived biopolymers have been used for various applications such as drug delivery, bioimaging, and tissue engineering.^7^,^8^ HSA is an abundant plasma protein responsible for retaining the colloid osmotic pressure and the solubilisation of lipophilic molecules in blood. It serves as a defined platform with high molecular weight (66.3 kDa) and distinct amino acid sequence that allow various post-modifications.^9^ The globular structure of HSA can be unfolded by protein denaturation followed by grafting polyethylene(oxide) (PEO) side chains that prevent uncontrolled aggregation and precipitation and retain the polypeptide main chain in solution. For stabilizing the denatured polypeptide backbone, several PEO chains were attached to thiol groups of HSA due to its hydrophilicity and more importantly, clinically proven biocompatibility by FDA.^10^ However, the chemical versatility of PEO is often limited by its scaffold and resultant functional group availability.

Herein, we introduce oligonucleotide sequences to replace PEO as a more efficient stabilization reagent that conserves the monodispersity of the system and offers opportunities for further functionalization (Scheme 1). The combination of a precise protein-derived polyamide backbone and stabilizing ssDNA chains yields copolymers of high molecular weights and monodispersity. The smart branches provide opportunities to position additional functionalities based on the accurate DNA hybridization with the grafted oligonucleotide side chains.^11^

**Scheme 1:**
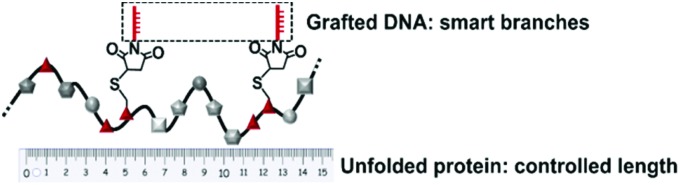
Protein-derived copolymer with distinct structure and smart side chains.

To graft DNA side chains, the globular structure of HSA was first unfolded in urea and the disulfide bridges were reduced by tris(2-carboxyethyl)phosphine (TCEP) to form the denatured polyamide backbone (dHSA) providing 35 free thiol groups originating from the cysteine residues (Fig. 1a). The ssDNA chains were attached to the dHSA backbone by applying a 15-mer ssDNA carrying a maleimide group at its 5′ terminal (maleimide-ssDNA,^12^ MW 4724 Da with MALDI-TOF spectrum in Fig. S1, ESI†) was selected and conjugated to the thiol groups *via* Michael addition under similar conditions as described before for PEO modification.^7^ In contrast to PEO, the 15-mer maleimide-ssDNA is sterically more demanding and provides 15 negative charges (originating from the sugar-phosphate backbone of the ssDNA) per ssDNA chain. These contribute to retaining the unfolded polypeptide backbone in aqueous media and efficiently preventing aggregation or precipitation. In addition, 15-mer sequences provide an adequate chain length for stable DNA hybridization (theoretical melting point: 65.1 °C 13) and acceptable yields during ssDNA solid phase synthesis.^14^ Thereafter, unreacted cysteine residues were capped by *N*-(2-aminoethyl)maleimide (Fig. 1a) to prevent disulfide formation and to improve shelf-life during storage.^7^

**Fig. 1:**
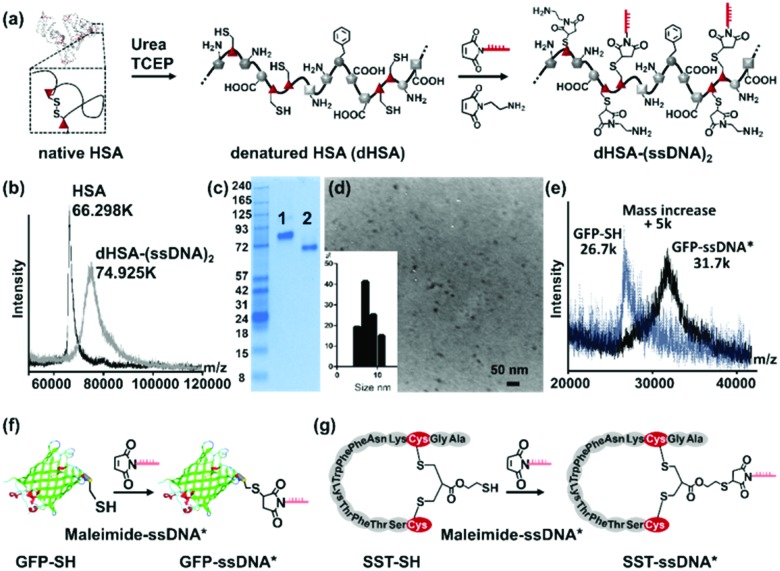
Synthesis of precision polymer dHSA-(ssDNA)_2_ and functionalization with complementary sequences. (a) Preparation of dHSA-(ssDNA)_2_ by reacting maleimide-ssDNA (5′-maleimide-CTCTACCACCTACTA-3′) with reduced cysteine residues of denatured HSA (dHSA); (b) MALDI-TOF spectra of dHSA-(ssDNA)_2_ (74.925k *m*/*z*) and native HSA (66.298k *m*/*z*) with normalized intensity; (c) native PAGE with HSA (lane 1), dHSA-(ssDNA)_2_ (lane 2, which was shifted to lower molecular weights due to higher negative surface charges); (d) TEM image of copolymer dHSA-(ssDNA)_2_ and the corresponding size distribution with an average radius of 9.05 nm; (e) MALDI-TOF MS spectra of GFP-SH and GFP-ssDNA* with normalized intensity; (f) GFP was conjugated to maleimide-ssDNA* (5′-maleimide-TAGTAGGTGGTAGAG-3′) yielding GFP-ssDNA*; (g) SST-SH was conjugated to maleimide-ssDNA* yielding SST-ssDNA*.

Purification of the reaction mixture was accomplished by ultrafiltration with a 30 kDa molecular weight cut-off membrane and followed by anion exchange chromatography.^15^ Notably, the number and conjugation position of the introduced ssDNA strongly affected the retention times during chromatography as well as the electrophoretic mobility in gel electrophoresis of the produced copolymers. This feature facilitated the purification of the product from the remaining reagents and complete separation of each fraction, which were not possible for the corresponding dHSA-PEO copolymers. After chromatography, seven fractions (UV absorbance spectra in Fig. S2a, ESI†) were collected, and analyzed by a 10% SDS-PAGE (Fig. S2b, ESI†) as well as MALDI-TOF MS (Fig. S2c and d, ESI†). Each copolymer fraction revealed a clearly distinguishable, discrete band on the gel in contrast to the “smeary bands” of polymers observed as dHSA-PEO (Fig. S2e, ESI†),^7^ indicating that copolymers of narrow dispersity with identical surface charges were collected in each fraction. The main product was obtained in the fraction 4 and used for all following experiments (more detailed analysis in ESI†). The MALDI-TOF MS spectrum revealed a sharp signal at 74.925 kDa (Fig. 1b) comparable to pure HSA (66.298 kDa), which is unique for synthetic polymers with small PDI and comparable to the largest dendrimers reported.^16^ According to the detected molecular weight, around two ssDNA chains were conjugated to the dHSA backbone yielding dHSA-(ssDNA)_2_ (calculated mass: 75.1 kDa) In contrast to the average attachment of 16 PEO chains (MW: 2 kDa) of previous studies^7^ that proved to be essential to retain the solubility of the copolymer in solution, surprisingly, only two ssDNA sequences were sufficient to stabilize dHSA. This was most likely due to their bulky architecture and a high number of negative charges.

After the attachment of two ssDNA chains, the zeta potential (Fig. S3a, ESI†) decreased from –7.6 ± 2.02 mV (native HSA) to –21.15 ± 0.63 mV (dHSA-(ssDNA)_2_. Consequently, in comparison to native HSA, the band for dHSA-(ssDNA)_2_ was shifted to lower molecular weights in the native PAGE according to the significantly enhanced number of negative surface charges (Fig. 1c). The morphology and size of dHSA-(ssDNA)_2_ was analyzed by transmission electron microscopy (TEM) and dynamic light scattering (DLS). In the dry state, nanometer-sized structures with an average dimension of 9.05 ± 2.0 nm and globular architectures were observed in the TEM image (Fig. 1d), whereas in solution, DLS measurements indicated macromolecular objects with a hydrodynamic radius of 17.9 ± 2.8 nm (PDI 0.22) for dHSA-(ssDNA)_2_ (Fig. S3d, ESI†). The dHSA-(ssDNA)_2_ retained the remarkable solubility of HSA since it could be re-dissolved easily after lyophilization. It should be noted that no larger aggregates were observed in all experiments conducted. Compared to PEO-stabilized copolymers (dHSA-PEO), dHSA-(ssDNA)_2_ provides two attachment points for the sequence-specific DNA hybridization, which encodes for rapid and site-specific conjugation of the desired functionalities containing the complementary ssDNA. Such template-based loading of functionalities resembles a LEGO®-type construction based on precise sequence recognition (Fig. 2a). To demonstrate the versatility of this approach, diverse functional groups such as (1) commercially available fluorescein chromophore labelled with complementary ssDNA (FITC-ssDNA*), (2) the green fluorescent protein (GFP) and (3) the peptide hormone somatostatin equipped with complimentary ssDNA* was loaded to the dHSA-(ssDNA)_2_. For ssDNA* conjugation, GFP was equipped with a single cysteine group by site-directed mutation of the glycine at position 51 into cysteine.^15^ Then, maleimide-functionalized complementary ssDNA (maleimide-ssDNA*, MW 5004 Da, MALDI-TOF in Fig. S4, ESI†) reacted with GFP following a published procedure^8^ yielding GFP-ssDNA* (Fig. 1f). The reaction was monitored by SDS-PAGE (Fig. S5a, ESI†) and MALDI-TOF MS, which indicated a mass increase of 5 kDa, attributed to the conjugated ssDNA* (Fig. 1e). The third functionality is a cyclic neuropeptide somatostatin (SST), binding to somatostatin cell membrane receptors (SSTR) that are overexpressed at the surface of various tumor cells and SST-mediated binding induces translocation into the cytosol.^17^ Thiolated SST (SST-SH) with preserved circular structure and SSTR binding activity, was obtained by re-bridging the disulfide bond with a linker molecule possessing a thiol group following a published protocol.^18^ SST-SH was reacted with the maleimide-ssDNA* to form SST-ssDNA* (Fig. 1g). The reaction was monitored by SDS-PAGE and MALDI-TOF spectra indicated an increase of 5 kDa, which was attributed to the conjugated ssDNA* (Fig. S6 and S7, ESI†).

**Fig. 2:**
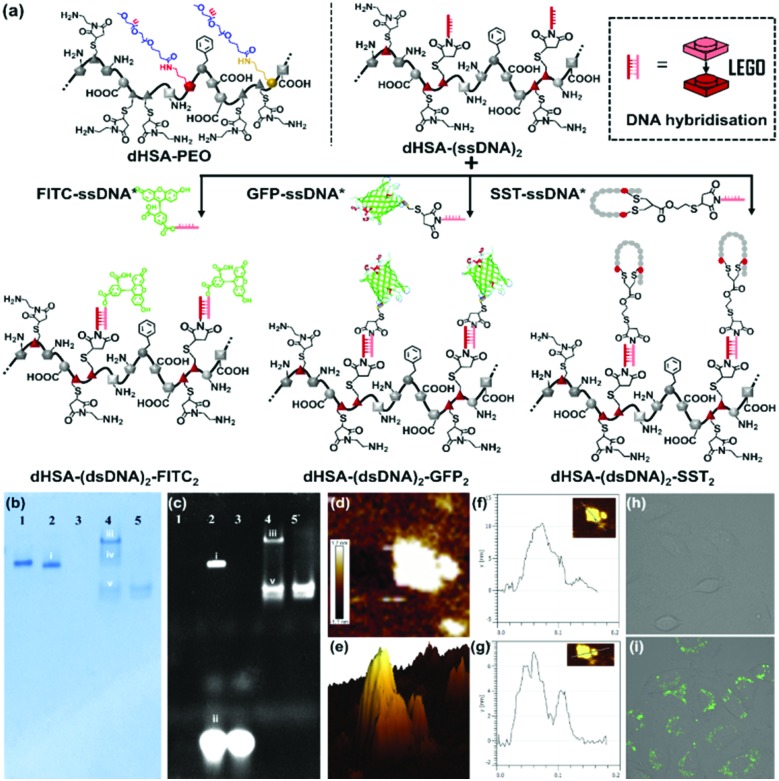
(a) Copolymer dHSA-(ssDNA)_2_ conjugated to different functionalities through DNA hybridization of FITC-ssDNA*, GFP-ssDNA* and SST-ssDNA*; (b and c) native PAGE of dHSA-(dsDNA)_2_-FITC_2_ and dHSA-(dsDNA)_2_-(GFP)_2_ after Coomassie Brilliant Blue staining, and fluorescent channel (lane 1, dHSA-(ssDNA)_2_; lane 2, band i, dHSA-(dsDNA)_2_-FITC_2_, band ii, FITC-ssDNA* residue; lane 3, FITC-ssDNA*; lane 4, band iii, dHSA-(dsDNA)_2_-GFP_2_, band iv, dHSA-(ssDNA)_2_ residue, band v, GFP-ssDNA* residue; lane 5, GFP-ssDNA*); (d–g) AFM image of dHSA-(dsDNA)_2_-GFP_2_ and height determination indicating three distinguishable peaks; (h and i) Confocal images of SST-mediated cell targeting. SST in dHSA-(dsDNA)_2_-SST_2_ mediates uptake into SSTR positive A549 cells as compared to dHSA-(ssDNA)_2_ as negative control in the fluorescein channel (normalized average fluorescence intensity is given in Fig. S10, ESI†).

Further incorporation of functionalities to dHSA-(ssDNA)_2_ was conducted by incubating dHSA-(ssDNA)_2_ (0.1 μg μL^–1^, 10 μL) in 1× TAE (Tris–acetate–EDTA) buffer with FITC-ssDNA* and GFP-ssDNA* (DNA molar ratio 1 : 10), to form dHSA-(dsDNA)_2_-FITC_2_ and dHSA-(dsDNA)_2_-GFP_2_, respectively (Fig. 2a). From PAGE analysis, the detected sharp product bands (Fig. 2b and c, Fig. 2b-i and iii), showing fluorescent signals, clearly indicated successful attachments of the corresponding FITC and GFP moieties to the copolymer and the formation of distinct products. The band shift in the native PAGE was influenced by both the increased negative surface charge (shown as faster shift in the gel), and the increased molecule size (shown as slower shift in the gel).^19^ Consequently, compared to the starting material dHSA-(ssDNA)_2_, the band i corresponding to dHSA-(dsDNA)_2_-FITC_2_ was shifted slightly higher due to the increased negative charges after hybridization with ssDNA*. In contrast, dHSA-(dsDNA)_2_-GFP_2_ revealed less band shift (band iii), which was attributed to the increased molecular mass of the product after loading of presumably two GFP proteins (GFP: MW 27 kDa). In this case, the retention due to the increase in molecular weight appeared more pronounced than the mobility due to ssDNA* hybridization. The height profile of the atomic force microscopy (AFM) images of dHSA-(dsDNA)_2_-GFP_2_ (Fig. 2d–g) revealed one major and two minor peaks. Using the information from the MALDI and gel electrophoresis the major peak with 7.2 nm in height was attributed to the random coil of dHSA-dsDNA as it fits well the dimensions of dHSA-(ssDNA)_2_. The two shorter peaks of about 4 to 6 nm heights were assigned to the two GFP proteins (more AFM pictures in Fig. S8, ESI†). The shift of the sharp band of dHSA-(ssDNA)_2_ before and after loading of the functionalities, the preserved fluorescence of loaded FITC and GFP in native PAGE, together with consistent macromolecular architecture observed from AFM indicate successful functionalization of the polyamide backbone through DNA hybridization.

Next, the peptide hormone somatostatin (SST) was attached to dHSA-(ssDNA)_2_ (labeled with fluorescein) using the same procedure as FITC/GFP-ssDNA* (Fig. 2a) and the resulting copolymer dHSA-(dsDNA)_2_-SST_2_ was characterized by agarose gel (Fig. S9, ESI†). The uptake of the prepared SST-loaded copolymer dHSA-(dsDNA)_2_-SST_2_ was tested in A549 cells, an adenocarcinomic human alveolar basal epithelial cell line that expresses SSTR.^20^ A549 cells were incubated with dHSA-(dsDNA)_2_-SST_2_ (44.5 nM) for 24 h to assess the cell targeting efficiency of the bioconjugate, whereas SST-deficient dHSA-(ssDNA)_2_ was applied as control. Confocal microscopy revealed an obvious increase in the cellular uptake of dHSA-(dsDNA)_2_-SST_2_, whereas no uptake was observed for dHSA-(ssDNA)_2_ as control (Fig. 2h and i). These experiments support that SST groups have been successfully attached to dHSA-(dsDNA)_2_ and retained their cell targeting bioactivity thus mediating the uptake of the negatively charged copolymer into SST receptor positive A549 cells.

DNA nanotechnology has provided the unique opportunity to design complex 2D to 3D nanostructures that serve as an assembly template.^21^ In this context, the functional and structural complexity of two ssDNA side chains conjugated to dHSA-(ssDNA)2 were further extended by applying multi-arm DNA linkers. ssDNA sticky ends could be placed at virtually any position of a DNA nanostructure to allow fast assembly of the desired functionalities encoded by the complementary DNA sequence.^22^ Thus, this template-based sequence recognition resembles a LEGO®-type 3D building block construction, which is fully extendable. To demonstrate the potential of this approach and to attach additional functionalities, a three-armed, chromophore labeled, Y shaped DNA linker (YDNA*)^23^ was designed by DNA nanotechnology encoding the sequence information to attach to the ssDNA of the polyamide main chain (Fig. 3a). As showed in Fig. 3b and c, the band of dHSA-(dsDNA-Y)_2_ in native PAGE became fluorescent after the addition of the YDNA* (labeled with Atto594 and Atto655) and was shifted to higher molecular weights. This shift was attributed to a higher contribution of the dragging effect of the increased molecular dimensions in contrast to the pushing effect of the negative charges of the loaded YDNA* (MW: 30.3 kDa). Again, the observed bands appear as discrete lines indicating the precise nature of the formed macromolecule. dHSA-(dsDNA-Y)_2_ with additional new binding motifs at each side chain provides now four ssDNA sequences for attaching two different functionalities (in total four) to the backbone. Thus, the Y-shaped linker serves as a branching point to further diversify the dHSA-(ssDNA)_2_ copolymer platform.

**Fig. 3:**
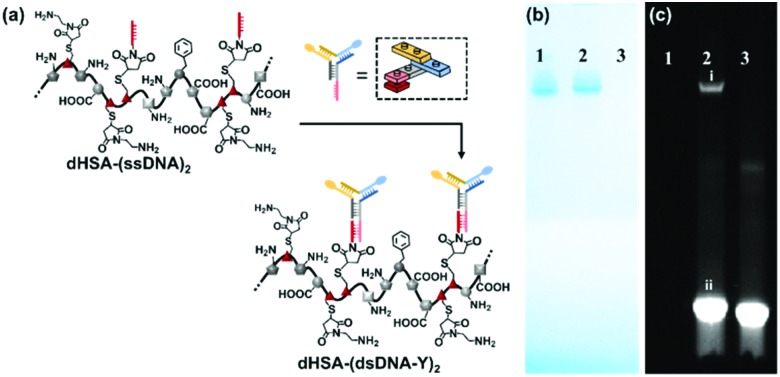
(a) ssDNA chain extension of the dHSA-(ssDNA)_2_ by Y shape DNA linker (termini labelled with Atto594 and Atto655); (b and c) Native PAGE of dHSA-(ssDNA)_2_ hybridization with YDNA*, measured by Coomassie Brilliant Blue staining and in the fluorescent channel (lane 1, dHSA-(ssDNA)_2_; lane 2, band i, dHSA-(dsDNA-Y)_2_, band ii, free YDNA*; lane 3 YDNA*).

In summary, dHSA-(ssDNA)_2_ with distinct structure and molecular mass was obtained by introducing ssDNA side chains to the denatured backbone of HSA. ssDNA exhibited high molecular mass, distinct monomer sequence and narrow dispersity due to the protein polyamide backbone and DNA side chains. A series of functionalities of different sizes and composition were conjugated to the complementary ssDNA and attached to dHSA-(ssDNA)_2_ by the DNA hybridization technique. A set of DNA-polypeptide hybrid polymers were prepared to support the robustness of our approach. Bioactivity was introduced by hybridization of the cyclic peptide hormone SST yielding dHSA-(dsDNA)_2_-SST_2_ that displayed high uptake into A549 cells that contain the somatostatin membrane receptors. In contrast, dHSA-(ssDNA)_2_ served as negative control that was not uptaken into A%$) cells. Furthermore, a Y-shaped DNA linker was successfully hybridized onto dHSA-(ssDNA)_2_ creating an additional branching point to further diversify functionalization as well as for the construction of larger 3D networks.^8^ The ssDNA side chains serve as versatile anchor groups for grafting distinct numbers of biologically active molecules under mild conditions and this approach might be applicable to other polymer systems as well. The combination of precise structures of DNA and protein precursor molecules and various functionalities, bioactive or fluorescent, along the polypeptide backbone and the ssDNA chains paves the way to precision therapeutics or theranostics.

Financial support by the ERC Synergy Grant No. 319130BioQ and the German Research Foundation, CRC 1279 (projects A5, C1 and C4) is gratefully acknowledged. We thank Mr Lennart Koepke for assistance in the gel experiments.

Open Access funding provided by the Max Planck Society.

## Conflicts of interest

There are no conflicts to declare.

## Supplementary Material

Supplementary information
