# Supplementary material for: Encoding function into polypeptide-oligonucleotide precision biopolymers
Source: Chem Commun (Camb). 2018 Sep 26;54(83):11797–800. doi: 10.1039/c8cc04725a (PMC6192144; doi:10.1039/c8cc04725a)
Supplement: Supplementary file 1 [file CC-054-C8CC04725A-s001.pdf]

Electronic Supplementary Material (ESI) for Chemical Communications.
This journal is © The Royal Society of Chemistry 2018

Encoding function into Polypeptide-oligonucleotide precision biopolymers Supporting information

Weina Liu, ‡[a,b] Felix Boldt, ‡[a,b] Yu Tokura,[a,b] Tao Wang,[b,c] Bikram Keshari Agrawalla,[a,b] Yuzhou Wu,*[a,d] Tanja Weil*[a,b]

[a]Max-Planck-Institute for Polymer Research, Ackermannweg 10, 55128 Mainz, Germany.

[b]Department of Inorganic Chemistry I, Ulm University, Albert-Einstein-Allee 11, 89081 Ulm, Germany.

[c]School of Materials Science and Engineering, Southwest Jiaotong University, 610031, Chengdu, China

[d]Hubei Key Laboratory of Bioinorganic Chemistry and Materia Medica, School of Chemistry and Chemical Engineering, Huazhong University of Science and Technology, Luoyu Road 1037, 430074 Hongshan, Wuhan, P.R. China

Material and methods

Copolymer dHSA-(ssDNA)$_2$ preparation and purification

1 mg native HSA was dissolved in degassed urea buffer (2 mL, 5 M urea, 50 mM phosphate buffer, 2 mM EDTA, pH 7.4) and stirred at room temperature for 15 min. TCEP (0.5 mg, 100 equivalent) was added as solid and the reaction mixture was stirred for additional 30 min under argon atmosphere. Then the maleimide terminated ssDNA (2.8 mg dissolved in 0.28 mL milli-Q water, 40 equivalent, synthesized, purified and deprotected according to literature[1]) was added to the denatured HSA solution and stirred under argon for 8 h. Thereafter, N-(2-Aminoethyl)maleimide (0.74 mg, 200 equivalent) was applied to cap the unreacted thiol group to avoid further cross linking between each dHSA polypeptide chain(Figure 1a). The native HSA, TCEP, N-(2-Aminoethyl)maleimide, and all the salt for buffer used in copolymer synthesis and purification are purchased from Sigma-Aldrich.

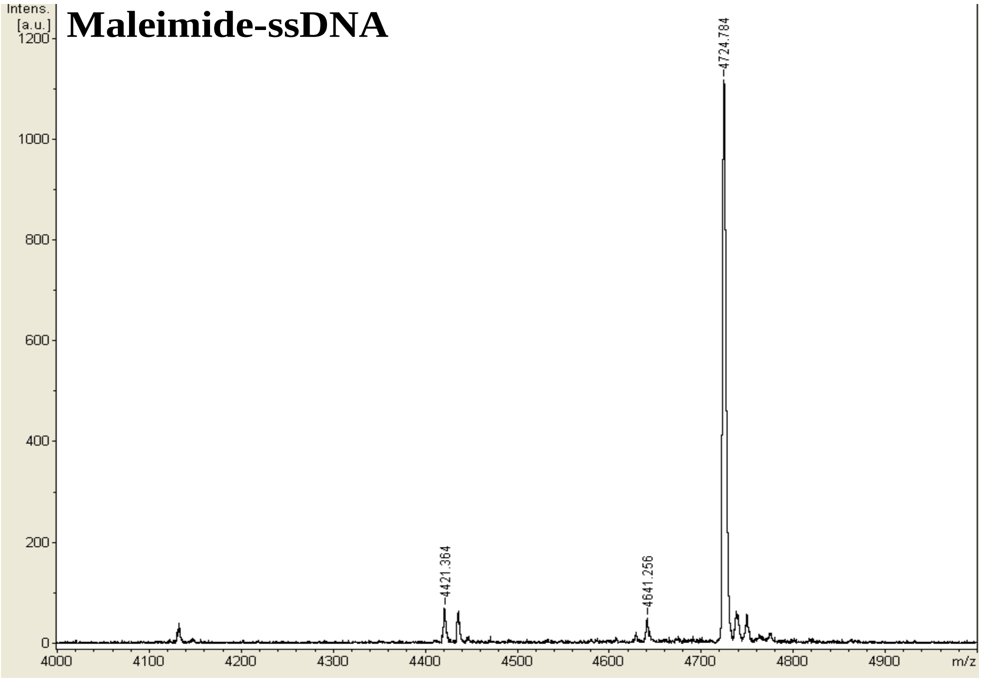

Figure S1, MALDI-TOF spectra of maleimide terminated ssDNA with protection group (maleimide-ssDNA, 5'-maleimide-CTCTACCACCTACTA-3', observed mass: 4724 Da, calculated mass: 4731 Da).

The reaction mixture was purified by ultrafiltration (30 KD molecular weight cut off) 5 times with urea buffer and 3 times with milli-Q water to remove the unreacted DNA and small molecules. Then the DNA stabilized dHSA was diluted to 1 mL in milli-Q water and loaded onto an anion exchange column (GE healthcare MonoQ 4.6/100 PE) mounted to fast protein purification system (GE healthcare Äkta purifier 10) (Buffer A 50 mM phosphate buffer, Buffer B 50 mM phosphate 1 M NaCl) to separate the macromolecules from the statistically protein-DNA conjugation mixture. All the seven fractions were collected and washed by ultrafiltration for 5 times to remove the salt, and preserved in 100 µL Mille-Q water. The 7 Fractions could be observed in the chromatogram (**F1-7**, Figure S2a). All fractions were analysed by SDS-PAGE gel (Figure S2b). **F7** could not be stained with coomassie blue and was therefore attributed to free DNA. All other fraction could be stained and where analysed with ImageJ software. The following ratios were obtained for each fraction: **F1**: 5.5%, **F2**: 1.7%, **F3**: 5.3%, **F4**: 49.0%, **F5**: 24.5%, **F6**: 14.1. **F1-3** was discarded because they only contain minor amount of sample. **F 4-6** were analysed by MALDI-TOF. Only **F4** showed a single peak, in contrast to **F5**, which showed a mixture of different compounds (Figure S2c and d), and **F6**, which could not be detected during the MLDI-TOF measurement at all. Therefore, **F4** was identified as main product and used for all following experiments. **F4** contained two bound DNA molecules as determined from the MALDI-TOF spectrum and was therefore referred to as dHSA-$(ssDNA)_2$. In the denatured SDS-PAGE (Figure S2b), the shift band of the copolymer is near 72KD compared to the protein maker, which is different from the MALDI-TOF result. The reason was attributed to the enhanced negative charge from the attached oligonucleotides, although the copolymers was denatured with SDS, the charge has still an effect on shift distance in the SDS-PAGE.

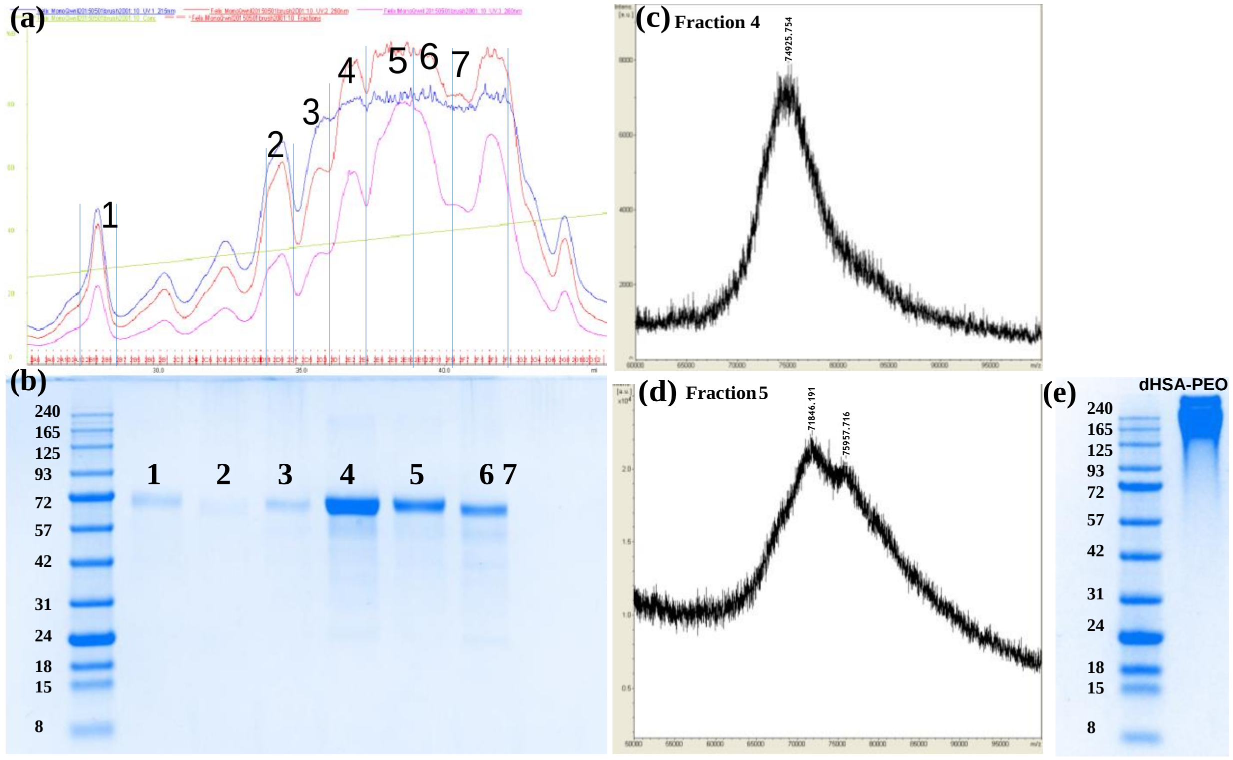

Figure S2, (a) UV absorbance for fractions 1-7 of protein-DNA copolymers with increased retention time on the anion exchange column; (b) SDS-PAGE of fraction 1-7 after purification; (c and d) MALDI-TOF spectra of fraction 4 and 5; (e) SDS-PAGE of dHSA-PEO with smeary band.

Characterization of the copolymers

MLDI-TOF measurement of the copolymers

All MALDI measurements were performed with a Reflex III equipped with a nitrogen laser (Bruker Daltonics). The machine was operated by flex control and the data was analysed by flex analysis.

The molecule weight of maleimide-ssDNA, maleimide-ssDNA*(Figure S1 and S4), and SST-ssDNA*(Figure S7) was measured by MALDI-TOF. Matrix: 95:10 mixture of 50 mg/ml 3-Hydroxy picolinic acid in 1:1 water: acetonitrile and 100mg/ml diammonium hydrogen citrate in water.

The molecule weight of SST-SH (Figure S6b) was measured by MALDI-TOF. Matrix: saturated a-cyano-4-hydroxy cinnamic acid 49,95:49,95:0,1 water: acetonitrile: trifluoracetic acid (v/v/v).

The molecule weight of GFP-SH and GFP-ssDNA*(Figure 1e) was measured by MALDI-TOF. Matrix: saturated sinapinic acid 49,95:49,95:0,1 water: acetonitrile: trifluoracetic acid (v/v/v) with triple layer (0.5 µL Matrix, 0.5µL sample, 2 µL Matrix).

The molecule weight of copolymer fraction 4 and 5(Figure S2c and d) was measured by MALDI-TOF. Matrix: Matrix used saturated a-cyano-4-hydroxy cinnamic acid 69,93:29,97:0,1 water: acetonitrile: trifluoroacetic acid (v/v/v).

Transmissions electron microscope imaging

A Jeol 1400 Transmissions electron microscope was used to measure the bright field TEM images of dHSA-$(ssDNA)_2$ (Figure 1d). The sample was prepared by dropping dHSA-$(ssDNA)_2$ (10 µg/mL, 5 µL dissolved in milli-Q water) on a freshly glow discharged 300 mesh size copper grid covered with a continuous carbon film and dried overnight at room temperature. Then the copper grid was stained by uranyl acetate.

Dynamic light scattering size and zeta potential measurement

The DLS size and zeta potential of dHSA-$(ssDNA)_2$ (Figure S3a and S3d, sample was dissolved in 10 mM KCl buffer, 100 µg/mL, 600 µL) was measured by a Malvern Zeta sizer ZEN3600 (Malvern Ltd, Malvern, UK) at 25°C with 173° angle.

Circular dichroism (CD) measurement

Circular dichroism was in measured to identify the change of molecular structure of the copolymer dHSA-$(ssDNA)_2$ (Figure S3b and c). As observed from the CD spectra, an increase in ordered α-helix and a decrease in β-sheet, β-turn and random coil elements was observed for the dHSA-$(ssDNA)_2$ copolymer in comparison to native HSA or dHSA-PEO as published before.[2] Denatured HSA (dHSA) could not be used as control as it precipitates from solution without the stabilizing side chains.

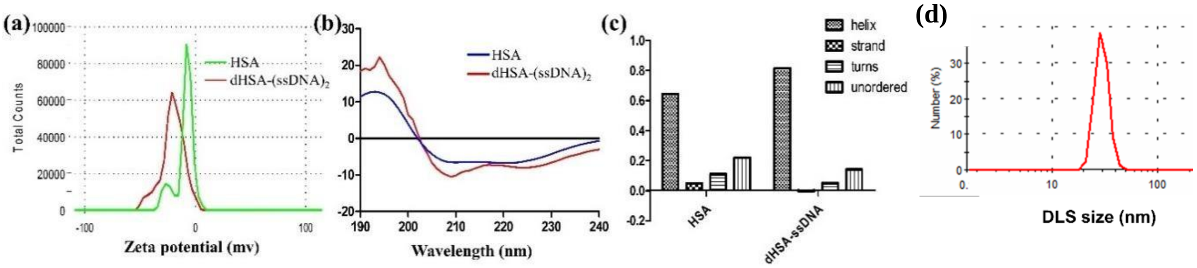

Figure S3. (a) Zeta potential of copolymer dHSA-$(ssDNA)_2$; (b and c) CD spectra and secondary structure distribution of copolymer dHSA-$(ssDNA)_2$ compared with native HSA; (e) DLS size of dHSA-$(ssDNA)_2$.

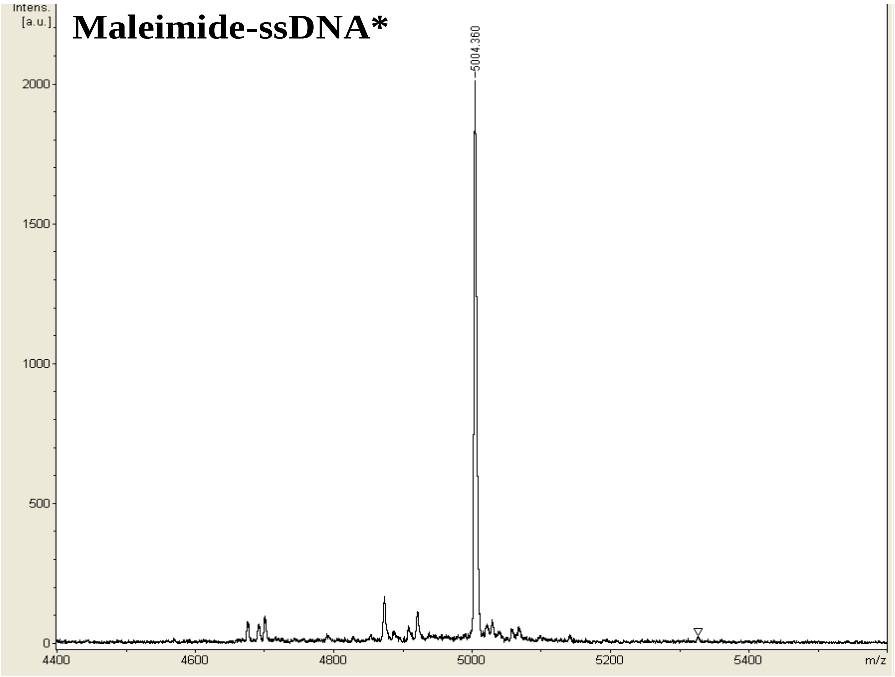

Figure S4, MALDI-TOF spectra of complementary sequenced, maleimide terminated ssDNA with protection group (Maleimide-ssDNA*, 5'-maleimde-TAGTAGGTGGTAGAG-3', observed mass: 5004 Da, calculated mass: 5011 Da).

Complementary sequence ssDNA* conjugated with functional molecules

FITC-ssDNA* conjugation

Fluorescein labeled complementary sequenced ssDNA* (FITC-ssDNA*) was purchased from Biomers GmbH, Ulm.

GFP-ssDNA* conjugation

GFP with a free cysteine group was expressed using the Q5 Site-Directed Mutagenesis Kit from New England BioLabs. The site-directed mutagenesis was performed according to manufactures protocol. The expressed GFP-SH (sequence in publication[3]) was purified by conventional His-Tag column mounted on a fast protein purification system (GE healthcare Äkta purifier 10). The GFP-SH and freshly deprotected maleimide-ssDNA* were mixed in an approximately equimolar ratio (exact ratio was predetermined for each batch) in milliQ water and incubated for 4 h on shaker at room temperature (400 RPM) to generate GFP-ssDNA* (Figure 1f). The reaction mixture was purified through anion exchange column (GE healthcare MonoQ 4.6/100 PE) mounted on a fast protein purification system (GE healthcare Äkta purifier 10) (Buffer A 50 mM phosphate buffer, Buffer B 50 mM phosphate 1 M NaCl) (UV absorbance spectra in Figure S5b). The GFP-SH and maleimide-ssDNA* conjugation as well as the purified GFP-ssDNA* was monitored by 10% native PAGE (Figure S5a). The isolated original GFP-SH as well as the purified GFP-ssDNA* were measured by MALDI-TOF to identify the mass (Figure 1e). The purified GFP-ssDNA* was stored in the purification condition (50 mM phosphate with 0.45 M NaCl, 55% of Buffer A and 45% of Buffer B).

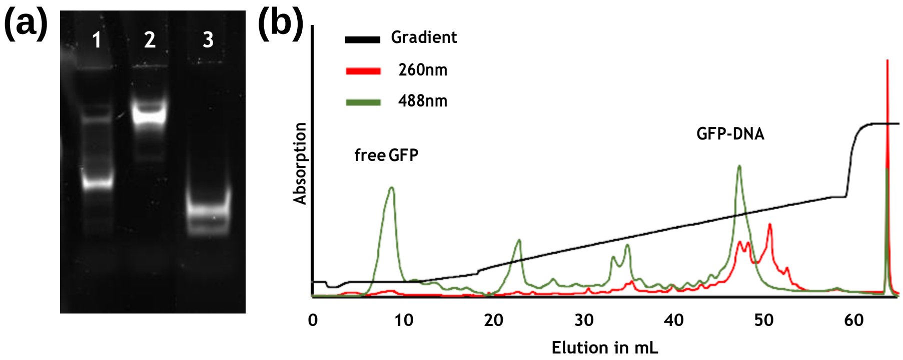

Figure S5. (a) SDS-PAGE gel of GFP-ssDNA* before and after purification, lane 1 reaction mixture of GFP-SH conjugated to Maleimide-ssDNA* to generate GFP-ssDNA* with slight free GFP-SH residue (upper band) and main product GFP-ssDNA*(lower band); lane 2, GFP-SH control; lane 3, GFP-ssDNA* after the column purification. The slightly shift of GFP-ssDNA* after purification was attribute to the

charge effect from the gradient buffer; (b) UV absorbance spectra for GFP-ssDNA* purification through anion exchange column.

SST-SH synthesis and SST-ssDNA* conjugation

Bis-alkylation intercalator reagent 2-((*tert*-butoxycarbonyl)thio)ethyl 2-(tosylmethyl)acrylate, which introduces a single thiol anchor group to generate thiolate somatostatin (SST-SH), by converting the chemically labile di-sulfide bond into two more stable bis-sulfides. The bis-alkylation intercalator and SST-SH was synthesized and purified from the published protocol[4] (Figure S6a).

The SST-SH was conjugated to maleimide-ssDNA* to generate SST-ssDNA*(Figure 1g). The SST-SH with maleimide-ssDNA* (1 and 2 equimolar ratio) were mixed in 10 mM $MgCl_2$ buffer, 10% Methanol and incubated for 4 h on shaker at room temperature (400 RPM). The outcome of the reaction, especially unreacted ssDNA* was determined by native PAGE with aluminum sulfate containing Coomassie Brilliant Blue according to a previously published protocol.[5] The conjugation efficiency of SST-ssDNA* was tested with native PAGE (Figure S6c) and the reaction mixture was purified through anion exchange column (GE healthcare MonoQ 4.6/100 PE) mounted on a fast protein purification system (GE healthcare Äkta purifier 10) (Buffer A 50 mM phosphate buffer, Buffer B 50 mM phosphate 1 M NaCl) (UV absorbance spectra in Figure S6d). The formed SST-ssDNA* (MALDI-TOF spectra in Figure S7) with MW 6701 Da was isolated and loaded to copolymer dHSA-$(ssDNA)_2$ by DNA hybridization.

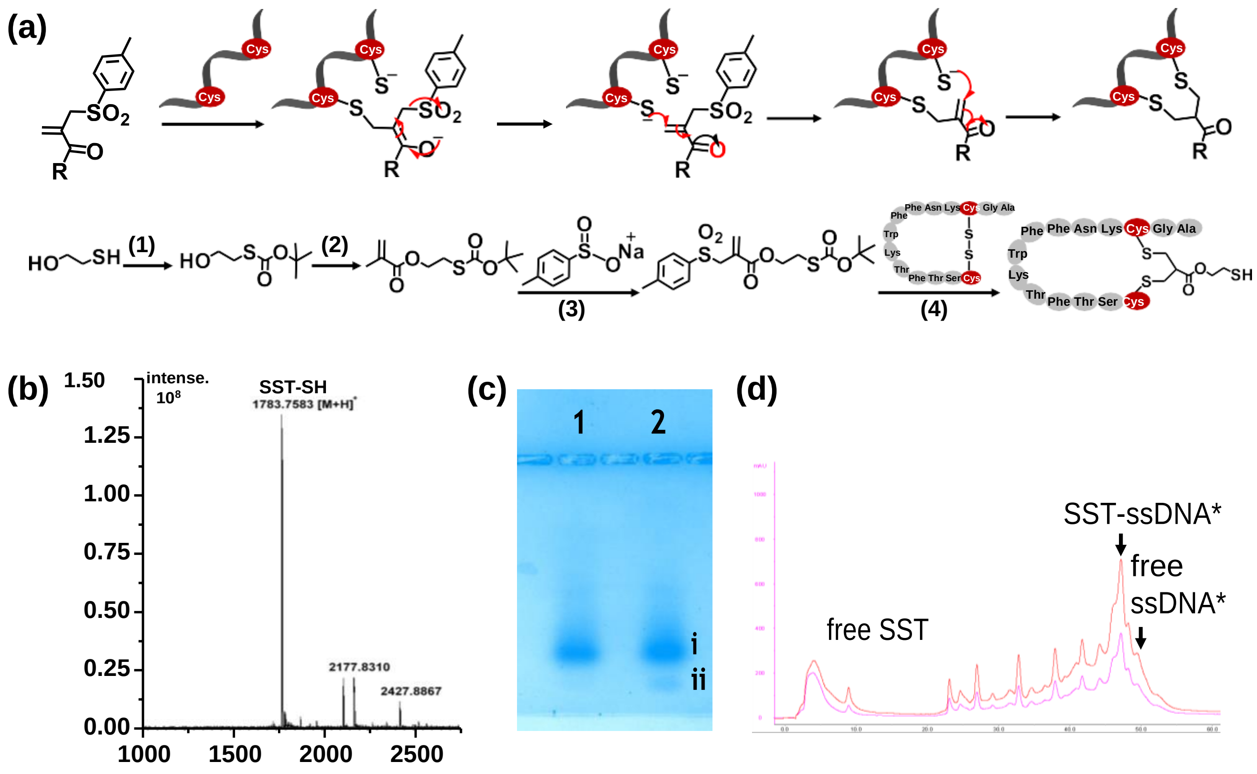

Figure S6, (a) Reaction mechanism of SST-SH synthesis;[4] (b) MALDI-TOF spectra of SST-SH (observed mass: 1783 Da, calculated mass: 1783 Da); (c) Agarose gel 1% of the SST-ssDNA* conjugation with 1 and 2 maleimide-ssDNA* equivalent (lane 1 and 2) and relatively high conjugation efficiency, band i SST-ssDNA*, band ii Maleimide-ssDNA* residue; (d) UV absorbance of SST-ssDNA* purification.

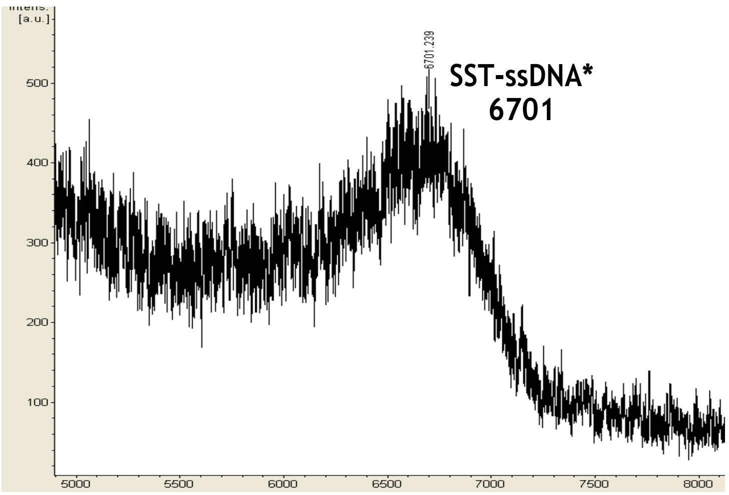

Figure S7, MALDI-TOF spectra of SST-ssDNA* (observed mass: 6701 Da, calculated mass: 6698 Da).

Functional molecule loading by non-covalent DNA hybridization

dHSA-(dsDNA)$_2$-FITC$_2$

FITC-ssDNA* (1 µg/µL, 0.6µL, 10 equivalent) was incubated with the copolymer dHSA-(ssDNA)$_2$ (100 µg/mL, 10 µL) in 1× TAE buffer (37°C, 500 rpm for 4 h incubation) to load to generate dHSA-(dsDNA)$_2$-FITC$_2$ (Figure 2a). The dHSA-(dsDNA)$_2$-FITC$_2$ reaction mixture was monitored by a 10 % Native-PAGE gel.

dHSA-(dsDNA)$_2$-GFP$_2$

GFP-ssDNA* (1 µg/µL in 50 mM phosphate with 0.45 M NaCl, 4.5 µL, 10 equivalent) was incubated with the copolymer dHSA-(ssDNA)$_2$ (100 µg/mL, 10 µL) with a final buffer concentration of 1 × TAE, 15.5 mM phosphate and 139 mM NaCl buffer (37°C, 500 rpm for 4 h incubation) to generate dHSA-(dsDNA)$_2$-GFP$_2$ (Figure 2a). The dHSA-(dsDNA)$_2$-GFP$_2$ reaction mixture was monitored by a 10 % Native-PAGE.

The morphology of dHSA-(dsDNA)$_2$-GFP$_2$ was measured by atomic force microscopy (FastScan Bio, Bruker Dimension) with ScanAsyst fluid+ cantilever and ScanAsyst mode for imaging (Figure S8). The sample preparation method was as following. The GFP loaded copolymer dHSA-(dsDNA)$_2$-GFP$_2$ (10 µg/mL, 70 µL dissolved in milli-Q water) was dropped on $MgCl_2$ buffer pre-incubated mica discs.

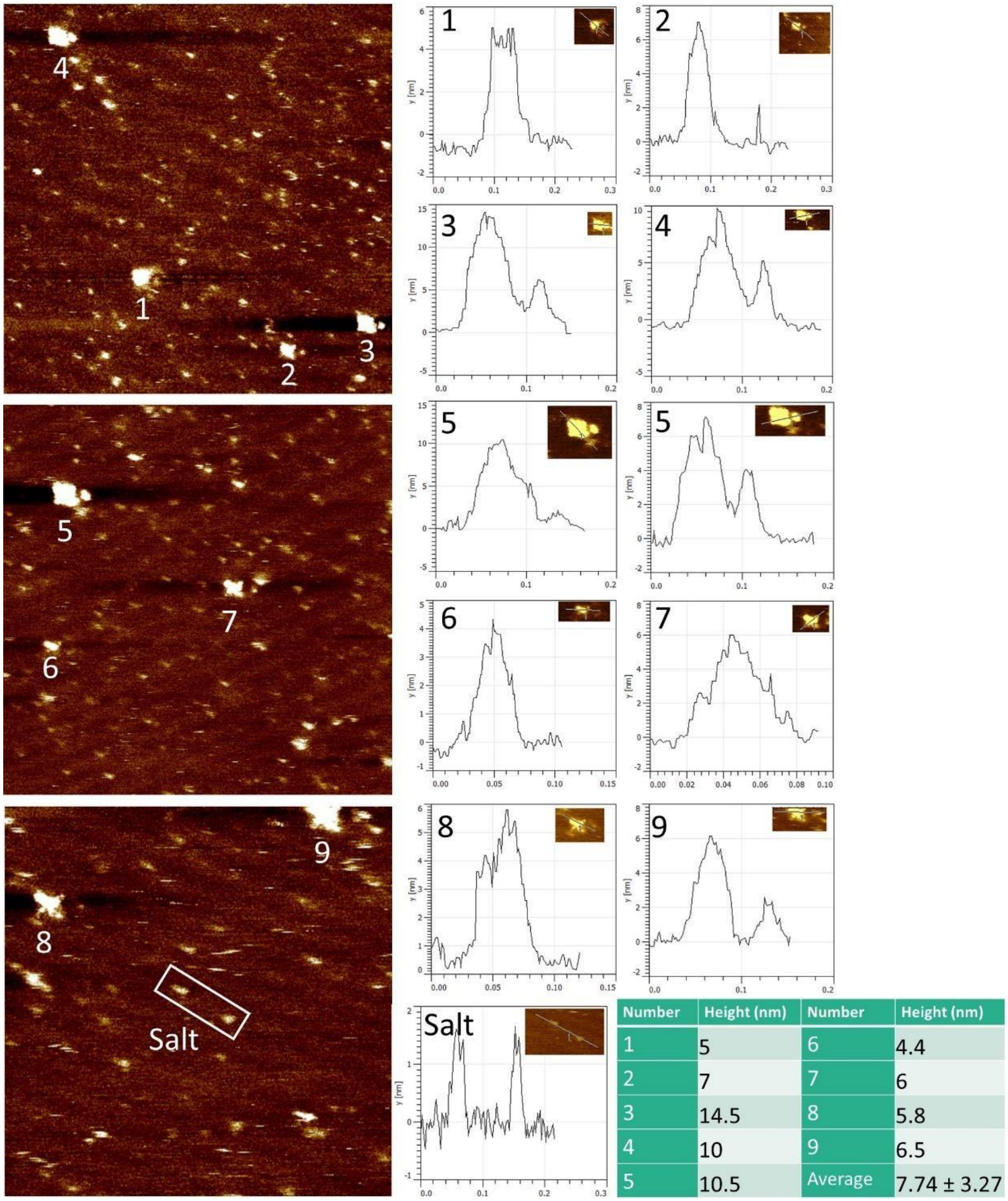

Number	Height (nm)	Number	Height (nm)
1	5	6	4.4
2	7	7	6
3	14.5	8	5.8
4	10	9	6.5
5	10.5	Average	7.74 ± 3.27

Figure S8, AFM image of dHSA-$(dsDNA)_2$-GFP_2. Nine of the copolymer macromolecule was measured statistically with an average height of 7.74 ± 3.27 nm. Most of those macromolecules have additional peaks or a shoulder of the main peak. Smaller sized morphologies with height of 1.5 nm and narrow width were considered as salt crystals or dust, since the formation condition of dHSA-$(dsDNA)_2$-GFP_2 was in concentrated buffer with 1 × TAE, 15.5 mM phosphate and 139 mM NaCl.

dHSA-(dsDNA)$_2$-SST$_2$

SST-ssDNA* (0.2 µg/µL, 0.84 µL, 2 equivalent) was mixed with FITC labeled dHSA-(ssDNA)$_2$ (100 µg/mL, 10 µL) and incubated on shaker (37 °C, 400 rpm for 4 h) to generate SST loaded copolymer dHSA-(dsDNA)$_2$-SST$_2$ (Figure 2a). Then the reaction mixture was analyzed by 3 % agarose gel electrophoresis. The new entity SST-ssDNA* contributed opposing effects to the shift of the entire construct. The increased size of the constructs results in a retardation of electrophoretic mobility while the additional charges increase the electrophoretic mobility. In this case both effects compensate each, therefore no band shift was obtained for newly generated dHSA-(dsDNA)$_2$-SST$_2$, but the success of the reaction was clearly observed by the broadening of the band and an increase band contrast of both commassie brilliant blue staining and fluorescence compared to an equal amount of dHSA-(ssDNA)$_2$ loaded onto another pocket (Figure S9a and b, lane 2 band i compared with lane 3).

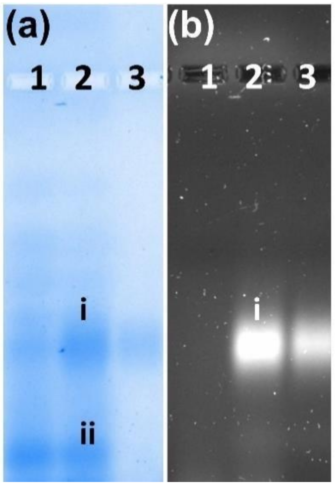

Figure S9, Agarose gel 3% of the conjugation SST-ssDNA* and FITC labelled dHSA-(ssDNA)$_2$ (lane 1, SST-ssDNA*; lane 2, SST-ssDNA* and dHSA-(ssDNA)$_2$ conjugation mixture (band i, dHSA-(dsDNA)$_2$SST$_2$ and free dHSA-(ssDNA) $_2$ mixture, band ii, free SST-ssDNA*); lane 3, dHSA-(ssDNA)$_2$ control.

Cell culture and confocal microscope imaging

The reaction mixture of FITC labelled dHSA-$(dsDNA)_2$-SST_2 (44.5 nM) was incubated with A549 cells for 24 h in incubator. Non-SST loaded dHSA-ss$(DNA)_2$ with FITC labelling (44.5 nM) was also incubated with A549 cells as negative control. The confocal imaging was performed by LSM 710 laser scanning confocal microscope system (Zeiss, Germany) coupled to an XL-LSM 710 S incubator and

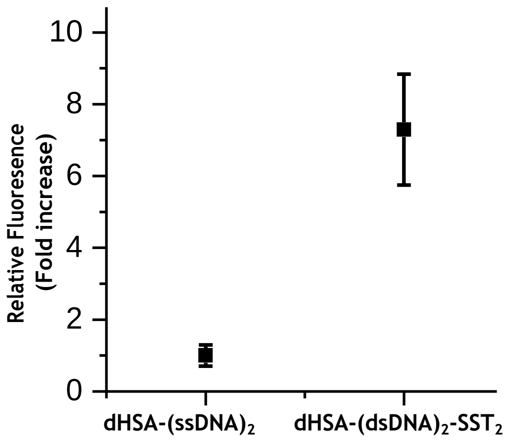

equipped with a 63× oil immersion objective.

Figure S10, Fluorescence intensity expressed as fold increase for cells treated with copolymers dHSA-$(ssDNA)_2$ and dHSA-$(dsDNA)_2$-SST_2. Figure is generated by ImageJ following published method.[6]

Y-shaped DNA* linker construction and loading to copolymer dHSA-$(ssDNA)_2$ for side ssDNA chain extension.

Y-shaped DNA* linker (YDNA*) was constructed by ssDNA S1, S2, S3 with half of the sequence complementary to each other. S1 was equipped with a sticky end, complementary to the ssDNA from the copolymer dHSA-$(ssDNA)_2$. S2 and S3 was labelled with chromophore Atto594 and Atto655 as modal functionalities, separately. The YDNA was constructed by ssDNA S1, S2 and S3 hybridization in 1× TAE buffer (S1 : S2 : S3 = 1.2 : 1 : 1). The reaction proceeded with high conjugation efficiency (Figure S11) and the YDNA was loaded to the copolymer dHSA-$(ssDNA)_2$ without any further purification.

The ssDNA S1, S2 and S3 were purchased from Biomers GmbH, Ulm.

S1 5'- CCT GTC TGC CTA ATG TGC GTC GTA AG T AGT AGG TGG TAG AG -3'

S2 5'-ATTO594 CTT ACG ACG CAC AAG GAG ATC ATG AG -3'

S3 5'- ATTO655 CTC ATG ATC TCC TTT AGG CAG ACA GG -3'

YDNA* (10 equivalent) was incubated with dHSA-$(ssDNA)_2$ (100 μg/mL, 10 μL) in 1× TAE buffer (37 °C, 500 rpm for 4 h) to generate YDNA* loaded copolymer dHSA-$(dsDNA\text{-}Y)_2$ (Figure 3a). The reaction mixture was analyzed by 10% native PAGE gel.

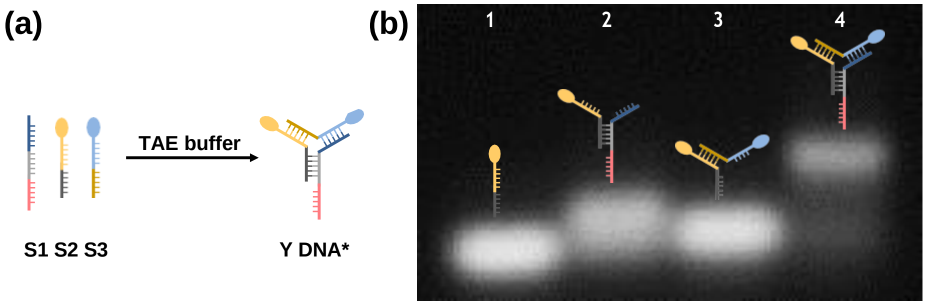

Figure S11, scheme and 3% agarose gel electrophoresis of Y shape linker DNA* construction, lane1, S2; lane 2, reaction mixture of S1 + S2; lane 3, reaction mixture of S2+S3; lane 4, reaction mixture of S1 + S2 + S3.

Reference:

1. A. Sánchez, E. Pedroso and A. Grandas, *Org. Lett.*, 2011, **13**, 4364-4367.
2. Y. Wu, S. Chakrabortty, R. A. Gropeanu, J. Wilhelmi, Y. Xu, K. S. Er, S. L. Kuan, K. Koynov, Y. Chan and T. Weil, *J. Am. Chem. Soc.*, 2010, **132**, 5012-5014.
3. Y. Wu, C. Li, F. Boldt, Y. Wang, S. L. Kuan, T. T. Tran, V. Mikhalevich, C. Fortsch, H. Barth, Z. Yang, D. Liu and T. Weil, *Chem. Commun.*, 2014, **50**, 14620-14622.
4. S. L. Kuan, T. Wang, M. Raabe, W. Liu, M. Lamla and T. Weil, *ChemPlusChem*, 2015, **80**, 1347-1353.
5. D. Kang, Y. S. Gho, M. Suh and C. Kang, *Bulletin-Korean chemical society*, 2002, **23**, 1511-1512.
6. R. A. McCloy, S. Rogers, C. E. Caldon, T. Lorca, A. Castro and A. Burgess, *Cell Cycle*, 2014, **13**, 1400-1412.
